# Bears don’t always mess with beers: Limits on generalization of statistical learning in speech

**DOI:** 10.3758/s13423-025-02690-w

**Published:** 2025-04-14

**Authors:** Timothy K. Murphy, Nazbanou Nozari, Lori L. Holt

**Affiliations:** 1 Waisman Center, University of Wisconsin-Madison, 1500 Highland Ave, Madison, WI 53705 USA; 2 Department of Surgery, University of Wisconsin-Madison, Madison, WI USA; 3 Department of Psychological and Brain Sciences, Indiana University, Bloomington, IN USA; 4 Cognitive Science Program: Indiana University, Bloomington, IN USA; 5 Department of Psychology, The University of Texas at Austin, Austin, TX USA

**Keywords:** Statistical learning, Speech perception, Speech production

## Abstract

Perception changes rapidly and implicitly as a function of passive exposure to speech that samples different acoustic distributions. Past research has shown that this statistical learning generalizes across talkers and, to some extent, new items, but these studies involved listeners’ active engagement in processing statistics-bearing stimuli. In this study, we manipulated the relationship between voice onset time (VOT) and fundamental frequency (F0) to establish distributional regularities either aligned with American English or reversed to create a subtle foreign accent. We then tested whether statistical learning across passive exposure to these distributions generalized to new items never experienced in the accent. Experiment 1 showed statistical learning across passive exposure but no generalization of learning when exposure and test items shared the same initial consonant but differed in vowels (*bear/pear → beer/pier*) or when they differed in initial consonant but shared distributional regularities across VOT and F0 dimensions (*deer/tear → beer/pier*). Experiment 2 showed generalization to stimuli that shared the statistics-bearing phoneme (*bear/pear → beer/pier*), but only when the response set included tokens from both exposure and generalization stimuli. Moreover, statistical learning transferred to influence the subtle acoustics of listeners’ own speech productions but did not generalize to influence productions of stimuli not heard in the accent. In sum, passive exposure is thus sufficient to support statistical learning and its generalization, but task demands modulate this dynamic. Moreover, production does not simply mirror perception: generalization in perception was not accompanied by transfer to production.

## Introduction

Encountering a talker with an idiosyncratic speaking style or a non-native accent can diminish speech comprehension (e.g., Bradlow & Bent, 2008). But experience often leads to improvements that generalize to other contexts (e.g., Xie & Myers 2017). Sometimes, such encounters even impact subtle characteristics of one’s own speech (e.g., Pardo et al., 2017). Although instances of such adaptation and convergence are well documented, many questions regarding their bases remain unanswered.

A literature examining dimension-based statistical learning provides a means with which to fill these gaps (e.g., Idemaru & Holt, 2011; Liu & Holt, 2015; Schertz et al., 2016; Wu & Holt, 2022). This work posits that subtle differences across talkers can be characterized as shifts in the underlying acoustic regularities – the statistical distributions – of speech. The somewhat different speech patterns of American English compared to Scottish English (Escudero, 2001), for example, can be modeled as distribution shifts across multidimensional acoustic space, and the impact of listening across these distributions on perception (as well as production) can be tracked.

Such distributional shifts can be studied experimentally. For example, Idemaru and Holt (2011) selectively sample *beer-pier* utterances across an acoustic space defined by voice onset time (VOT, the timing of articulators’ release vs. voicing onset) and fundamental frequency (F0, related to pitch). A Canonical sampling mirrors the F0xVOT distributions typical of American English: utterances with short VOT tend to have low F0 and be heard as/b/whereas those with long VOT tend to have higher F0 and be heard as/p/. American English adults’ perception mirrors these regularities, with VOT serving as a strong cue to/b/-/p/category identity and F0 contributing to a lesser extent (Wu & Holt, 2022). Reversing this correlation creates a subtle accent. In a passive exposure version of the paradigm (Hodson et al., 2023; Murphy et al., 2024), listeners hear a sequence of *beer* and *pier* utterances conveying one of these distributional regularities followed by one of two F0-differentiated test stimuli with ambiguous VOT. With only F0 available to convey category identity, test stimulus categorization indexes listeners’ reliance on F0 in speech categorization. In a pattern now well replicated across many studies, F0 robustly signals *beer* versus *pier* when distributions mirror American English norms but F0 reliance is markedly reduced in the context of the accent (Holt, 2025)*.* This points to implicit learning of statistical speech regularities that has an immediate influence on the mapping of acoustics to speech, thus informing how listeners adapt to idiosyncratic or accented speech.

Generalization has been a valuable tool in examining the grain of representation across which this learning operates. For example, if learning operates across talker-specific representations, there should be no generalization to talkers not experienced in the accent. However, learning does generalize to new talkers (Liu & Holt, 2015). Likewise, generalization is evident across lexical items. For example, Idemaru and Holt (2020) report generalization across word contexts with differing vowels (*beer-pier* → *bear-pear*, and vice versa) and differing vowel-consonant frames (*beer-pier* → *bill-pill)*, although generalization effects were weaker than effects for the token experienced in the accent (see also Liu & Holt, 2015; Lehet & Holt, 2020; Zhang et al., 2021). In contrast, generalization is not apparent across the acoustic dimensions that convey speech regularities, like F0 and VOT. Idemaru and Holt (2014) find that *beer-pier* learning does not generalize to influence *deer-tear* although each samples a similar F0xVOT acoustic space.

Collectively, these studies point to phoneme-sensitive learning. However, in contrast to the passive exposure paradigm described above (Hodson et al., 2023; Murphy et al., 2024), generalization studies have relied exclusively on active tasks with overt, trial-by-trial categorization of both statistics-bearing “exposure” speech stimuli and the “test” stimuli that measure statistical learning and subsequent generalization. Correspondingly, in these prior studies, the response set includes responses that match the statistics-bearing speech (e.g., *bear-pear*) as well as responses to test generalization (*beer-pier*). This might be important. Wu and Holt (2022) observe that individual differences in the strength of category activation – as indexed by categorization accuracy for statistics-bearing exposure stimuli – predict the magnitude of down-weighting of F0 upon introduction of the accent. If active categorization were to more robustly drive category activation than mere exposure, there may be task-driven learning and/or generalization outcomes. Hodson et al. (2023) examined this possibility, finding common patterns of F0 down-weighting for active trial-by-trial categorization of exposure stimuli and passive exposure to them. Yet there remains an open question: is active categorization across statistics-bearing stimuli necessary for generalization? This paper tackles this question.

Experiment 1 examines generalization of statistical learning across passive exposure with a single response set (*beer-pier)* across all conditions. Listeners hear a sequence of utterances conveying canonical or reverse distributions, then categorize sequence-final, F0-differentiated *beer-pier* test stimuli across three conditions: No Generalization (*beer-pier* → *beer-pier*), same Phoneme Generalization (*bear-pear → beer-pier,* for which active categorization paradigms observe generalization), and same Dimension Generalization (*deer-tear* → *beer-pier,* for which no generalization is observed in active tasks). To anticipate the results, we replicate the null effect in the Dimension Generalization condition. But, unlike past studies, we do not observe generalization in the Phoneme Generalization condition in Experiment 1. In Experiment 2 we examine whether this difference arises from learning differences across passive exposure. We focus our investigation on exposure and test stimuli that share initial and final phonemes, and introduce a mixed response set (*beer-pier+bear-pear)* in the critical condition. The mixed response set restores generalization, despite the passive exposure.

As a secondary measure in each experiment, we elicit speech productions to attempt to replicate recently reported transfer of statistical learning from perception to production (Murphy et al., 2024) and to examine generalization in production. To foreshadow, we robustly replicate Murphy and colleagues (2024) for statistics-bearing stimuli: the learning arising with perceptual experience with an accent transfers to impact listeners’ speech productions. Intriguingly, this transfer is limited to stimuli heard in the accent. Generalization in perception is not reflected in production.

## Experiment 1

### Methods

**Fig. 1:**
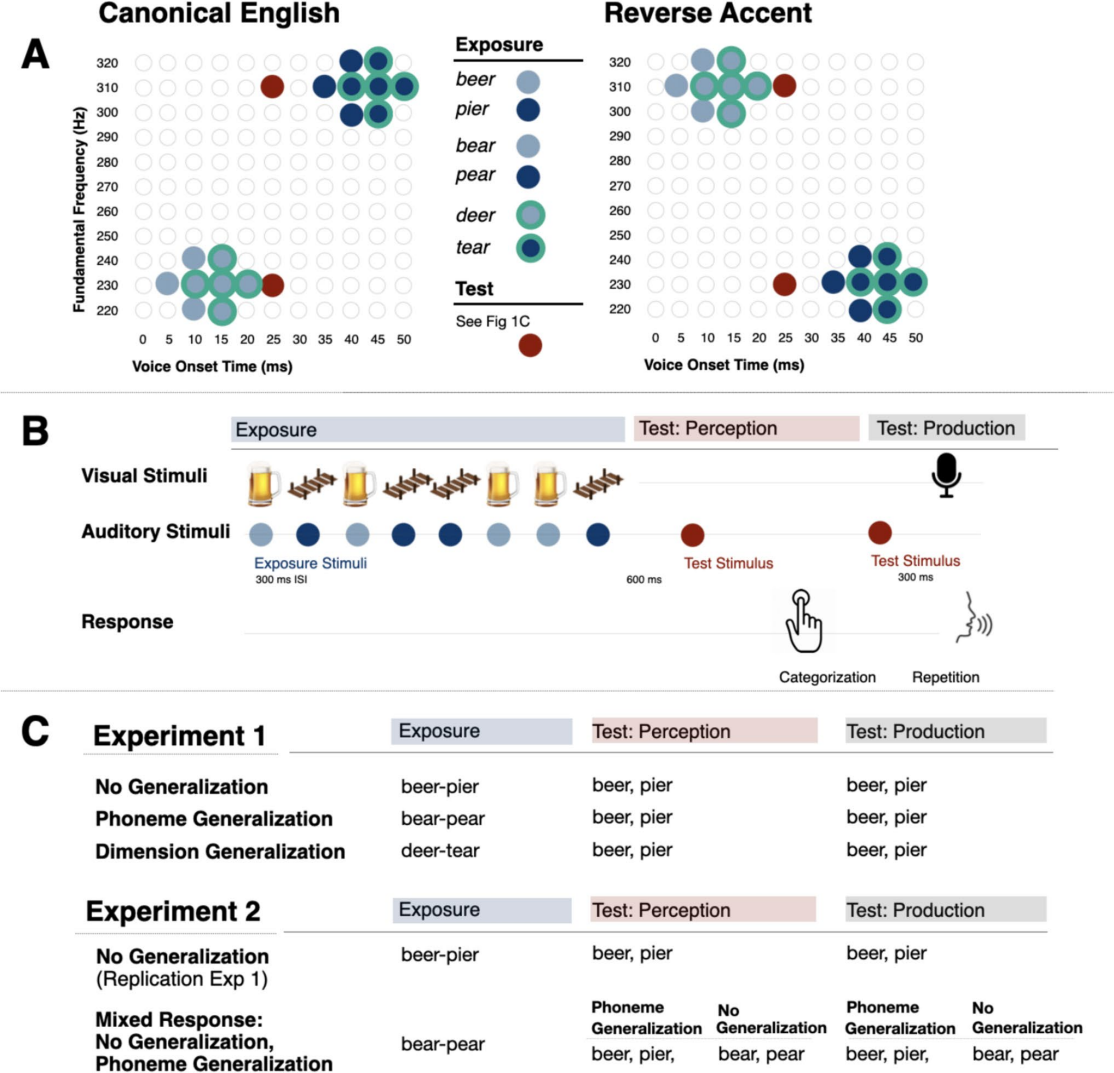
Experiment protocol**. A. Stimuli.** An acoustic space defined by voice onset time (VOT) and fundamental frequency (F0) conveyed *beer-pier* and *bear-pear* (solid blue, no line) and *deer-tear* (solid blue, aqua line) tokens sampled in a manner Canonical of American English or Reversed to convey an accent. **B. Trial structure.** A representative trial from the Experiment 1 Control condition illustrates the trial structure across each experiment, and all groups. **C. Experiment conditions.** The speech tokens that convey the short-term speech regularity (Exposure, blue) and the test stimuli that elicit perception (Perception, red) and production (Production, gray) are depicted for each condition of each experiment

Experiment 1 examined statistical learning across passive exposure to speech regularities and its generalization. Participants listened to a sequence of speech tokens possessing a (Canonical, Reverse) short-term distributional regularity and reported whether a final test stimulus was *beer* or *pier.* They then heard the same test stimulus again and repeated it aloud (Fig. 1). Test stimuli were always *beer-pier*, differentiated only by F0. Across conditions experienced by all listeners, the stimuli that conveyed distributional regularities across passive exposure varied: *beer-pier* (requiring No Generalization), *bear-pear* (Phoneme Generalization), *deer-tear* (Dimension Generalization).

### Participants

In keeping with past studies, we assumed a small effect size of d = 0.3 for generalization in speech perception (Idemaru & Holt, 2020; Liu & Holt, 2015). A power analysis performed using the program PANGEA (Westfall, 2015) indicated that a sample size of 90 participants would provide power > 0.8 to detect a three-way interaction between Test Stimulus F0, Canonical/Reverse statistical regularity, and the three-level generalization factor, at α = 0.05. As a provision against data loss in online studies, we collected online data from 110 adult (55 females) native English-speaking participants located in the USA. Eighteen participants’ data did not enter into analyses due to silent or highly noisy production recordings that precluded acoustic analysis of speech productions (N = 17) or perceptual responses indicating task noncompliance (N = 1). Data from 92 participants (49 females, mean age 28.1 years, SD = 4.8 years) entered the analysis.

### Stimuli

Figure 1A illustrates the speech stimuli. Fundamental frequency (F0) and voice onset time (VOT) varied, with other acoustic dimensions held constant, to create perceptual spaces corresponding to *beer-pier, bear*-*pear*, and *deer*-*tear.* Each of the six target words was spoken by an adult female native American English speaker, with specific tokens chosen to have similar duration (400 ms for *beer-pier* and *deer/tear*, 500 ms for *bear-pear*) and F0 contour. Beginning with these natural speech exemplars, we edited in the time domain to create 5-ms VOT steps (McMurray & Aslin, 2005). Next, we manipulated the F0 onset of each of these stimuli using a custom Praat script (Praat 6.1; Boersma & Weenink, 2023) such that onset F0 varied from 220 to 320 Hz in 10-Hz steps, with F0 contour interpolated smoothly across voicing to word offset. Amplitude normalization assured each stimulus possessed the same root mean-squared amplitude.

Exposure stimuli (blue, Fig. 1A) sub-sampled these acoustic spaces to create distinct short-term speech regularities. The Canonical English sampling (Fig. 1A, left) followed acoustic speech regularities typical of American English: stimuli with shorter VOT (< 25 ms) tend to have lower F0 and be labeled as/b/or/d/(light blue) whereas those with longer VOT (> 25 ms) tend to have higher F0 and be labeled as/p/or/t/(dark blue). A statistically defined “accent” reversed this distributional relationship from American English norms (Fig. 1A, right). Here, for the Reverse condition, shorter VOTs signal/b/or/d/but F0 is *higher* frequency. Longer VOTs signal/p/or/t/but F0 is *lower* frequency. *Beer-pier* and *bear-pear* tokens (blue, no line) shared identical F0xVOT values whereas *deer-tear* tokens (blue, aqua line) sampled distributions shifted + 5 ms in VOT to account for natural English VOT patterns (Cho & Ladefoged, 1999).

Additionally, two test stimuli possessed a perceptually ambiguous, 25-ms VOT and varied only in F0 (230 or 310 Hz; Fig. 1A, red symbols). Test stimulus categorization measured listeners’ reliance on F0 in category decisions related to learning (when test stimuli match exposure stimuli, e.g., *beer-pier → beer-pier*) and generalization (*bear-pear → beer-pier* and *deer-tear → beer-pier*). These same test stimuli elicited speech productions in the auditory repetition task. Exposure and Test stimuli were chosen on the basis of responses provided by nine raters and had been previously shown to drive statistical learning in perception (Murphy, 2024).

#### Procedure

Online participants recruited via Prolific.co were automatically directed to an experiment hosted on Gorilla (www.gorilla.sc, Anwyl-Irvine et al., 2021). Using the Chrome browser on a computer (no mobile devices), participants provided consent, completed a demographics survey, and underwent both a brief check of headphone compliance test (Milne et al., 2021) and a check that the computer microphone was recording utterances.

Figure 1B shows the trial structure. Participants listened passively to a sequence of eight perceptually unambiguous exposure stimuli that conveyed either a Canonical or a Reverse short-term regularity. Each sequence included four tokens from each of the two distributions (Fig. 1A, dark and light blue symbols), randomly selected and concatenated with 300-ms silent intervals separating utterances. Clipart images corresponding to the word expected from the perceptually unambiguous VOT appeared at the onset of each sound. Next, after 600 ms, participants heard one of the two test stimuli (High or Low F0; Fig. 1A, red symbols) and categorized it as *beer* or *pier* via a keyboard response with on-screen text to guide the mapping. Then, 300 ms later, the same test stimulus played again, and an image of a microphone prompted participants to repeat the word aloud. Participants had 2,500 ms to repeat the test stimulus and utterances were saved digitally for subsequent acoustic analysis of F0.

As summarized in Fig. 1C, *beer-pier* test stimuli elicited perceptual categorization responses and speech productions across each of three conditions. The statistics-bearing exposure stimuli of the No Generalization condition matched the *beer-pier* test stimuli, thereby measuring statistical learning without requiring generalization. In contrast, *bear-pear* exposure sequences in the Phoneme Generalization condition necessitated generalization of statistical learning to *beer-pier* test stimuli sharing a common initial phoneme. Finally, in the Dimension Generalization condition, the *deer-tear* regularities differed in initial phoneme from the *beer-pier* test stimuli but overlapped across F0xVOT acoustic dimensions.

For each condition, participants experienced 30 Canonical trials followed by 30 Reverse trials. Among these, 24 of 30 stimuli involved exposure stimuli followed by one of the two *beer-pier* VOT-ambiguous, F0-differentiated test stimuli described above; responses to these stimuli entered analyses. Six additional VOT-unambiguous stimuli served as a data-quality check of online participants, with a priori exclusion of participants who gave the same response to these unambiguous stimuli (no participants were excluded on this basis). Unambiguous *beer, bear,* and *deer* stimuli had a 230-Hz F0 and 10-ms VOT (*deer*: 15-ms VOT) while unambiguous *pier, pear,* and *tear* stimuli had a 310-Hz F0 and 40-ms VOT (tear: 45-ms VOT). As in prior studies (Wu & Holt, 2022), categorization of these perceptually unambiguous stimuli was consistent with expectations from English (Long VOT, 96%/p/; Short VOT, 93%/b/). A Latin square design assured balanced presentation of conditions across participants.

#### Statistical analyses

**Perceptual categorization.** We modeled the influence of statistical learning on perceptual categorization of test stimuli using mixed-effects models (*lme4*; Bates, Mochler, Bolker, & Walker, 2015) in R (version 4.1.3, R Core Development Team, 2022) with the binary (*beer, pier*) categorization response as the dependent variable. The full statistical model involved fixed effects across Statistical Regularity (Canonical, Reverse), Test Stimulus F0 (Low F0, High F0), and Condition (No Generalization, *beer-pier*; Phoneme Generalization, *bear-pear*; Dimension Generalization, *deer-tear*) as well as two- and three-way interactions. Random effects included by-subject random intercepts and random slopes for Statistical Regularity and Test Stimulus F0 over subjects. Statistical Regularity and Test Stimulus F0 fixed effects were center coded (− 0.5 or 0.5). A simple-effects coding scheme was applied to the three-level Condition effect whereby the No Generalization condition served as the reference level to which the Phoneme Generalization and Dimension Generalization conditions were compared. Three-way interactions among Statistical Regularity, Test Stimulus F0, and Condition were examined with post hoc tests of the Statistical Regularity by Test Stimulus F0 interaction for each Condition. Satterthwaite approximates using the *LmerTest* package (version 3.1–3, Kuznetsova, Brockhoff, & Christensen, 2017) provided *p* values.

**Speech production.** Transfer of statistical learning in listening to repetition productions was modeled across by-participant z-score normalized utterance F0 (as in Murphy et al., 2024). In brief, the F0 (computed across the first 40 ms) was measured for each utterance. F0 values ± 3 standard deviations from a participant’s mean F0 were removed from analysis. Next, we normalized F0 on a by-individual basis to account for F0 variability arising across talkers (Titze, 1989). Therefore, for production analyses, a z-score of 0 indicates the mean F0 for a participant across all productions. Positive and negative z-scores correspond to continuous standard deviation units above and below the mean, respectively, that we submitted to standard linear effects models. Fixed and random effect structures, and the approach to post hoc tests, were identical to perceptual statistical learning analyses.

## Results

### Perceptual categorization

**Fig. 2:**
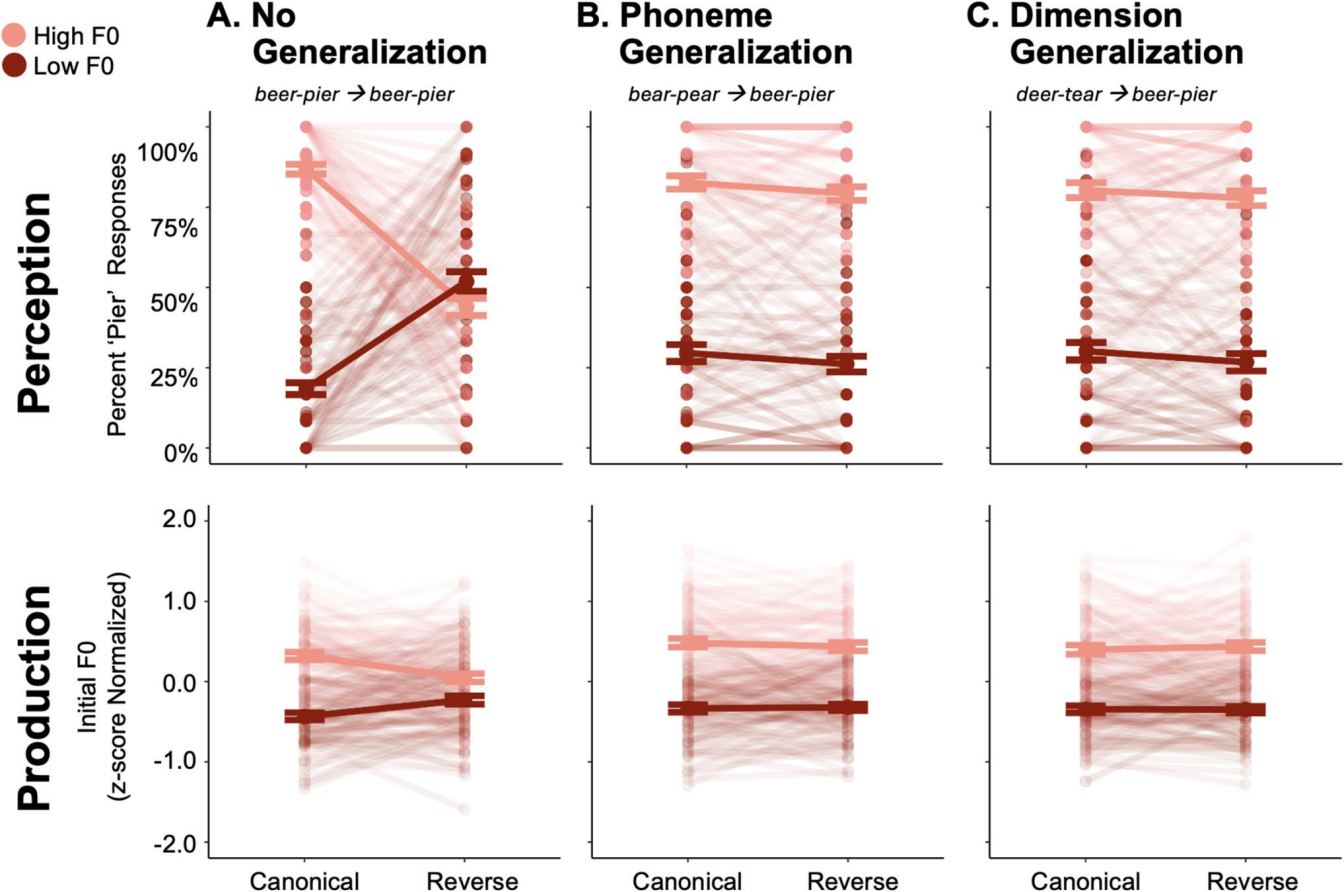
Experiment 1 perception and production results. The top row depicts percent *pier* categorization responses to High and Low F0 *beer-pier* test stimuli in the context of Canonical and Reverse short-term regularities. The bottom row shows z-score normalized fundamental frequency (F0) of *beer-pier* speech productions elicited in repetition of High and Low F0 test stimuli in the context of Canonical and Reverse short-term regularities. **A.** No Generalization (*beer-pier* exposure, *beer-pier* test) **B.** Phoneme Generalization (*bear-pear* exposure, *beer-pier* test). **C.** Dimension Generalization (*deer-tear* exposure, *beer-pier* test). Larger symbols and thick lines represent sample mean and standard error. Smaller symbols and transparent lines indicate individual participants’ behavior

Figure 2 presents perceptual categorization of F0-differentiated *beer-pier* test stimuli as a function in Canonical and Reverse conditions. Table 1 displays results of a logistic mixed effects model fit to these data.

**Table 1:** Experiment 1 perceptual categorization of test stimuli across conditions

	*Β*	*SE*	*z*	*p*
Intercept	0.22	0.08	2.74	0.006
Statistical Regularity	0.24	0.05	4.48	< 0.001
Test Stimulus F0	2.40	0.14	17.53	< 0.001
Phoneme Generalization	0.24	0.06	4.22	< 0.001
Dimension Generalization	0.18	0.06	3.17	0.002
Statistical Regularity x Test Stimulus F0	1.45	0.09	15.43	< 0.001
Statistical Regularity x Phoneme Generalization	− 0.10	0.11	− 0.85	0.396
Statistical Regularity x Dimension Generalization	− 0.14	0.11	− 1.28	0.199
Test stimulus F0 x Phoneme Generalization	1.01	0.11	8.97	< 0.001
Test stimulus F0 x Dimension Generalization	0.83	0.11	7.48	< 0.001
Statistical Regularity x Test Stimulus F0 x Phoneme Generalization	− 4.20	0.22	− 18.72	< 0.001
Statistical Regularity x Test Stimulus F0 x Dimension Generalization	− 4.28	0.22	− 19.27	< 0.001

Reference levels are Statistical Regularity (Reverse), Test Stimulus F0 (Low F0), Condition (No Generalization). Phoneme Generalization and Dimension Generalization result from simple effects coding comparing the respective conditions to the No Generalization condition

Across all conditions, there were more *pier* responses for High F0, as is typical in American English (Lisker, 1986), reflected in a main effect of Test Stimulus F0 (z = 17.52, *p* < 0.001) and a main effect of Statistical Regularity (z = 17.53, *p* < 0.001). Importantly, these factors significantly interacted (z = 15.43, *p* < 0.001), indicating that statistical learning across passive listening impacted reliance on F0 in categorization.

Simple-effects coding comparing perceptual responses from the No Generalization condition to responses from the Phoneme Generalization and Dimension Generalization revealed significant main effects (Phoneme: z = 4.22, *p* < 0.001; Dimension: z = 3.17, p = 0.002) indicative of an overall difference in perceptual response across conditions. Two-way interactions were significant between each condition and Test Stimulus F0 (Test Stimulus F0 by Phoneme: z = 8.97, *p* < 0.001; Test Stimulus F0 by Dimension: z = 7.48, *p* < 0.001) but not Statistical Regularity. Importantly, two significant three-way interactions indicated that perceptual down-weighting differs for both the Phoneme Generalization (z = − 18.70, *p* < 0.001) and Dimension Generalization (z = − 19.27, *p* < 0.001) conditions relative to the No Generalization condition.

Based on these two significant three-way interactions, we tested statistical learning/generalization in each condition with separate, post hoc logistic mixed effect models. The two-way interaction between Test Stimulus F0 and Statistical Regularity was significant only in the No Generalization model (z = 23.72, *p* < 0.001), but not the Phoneme (z = 0.57, p = 0.567) or Dimension (z = 0.94, p = 0.348) Generalization models. Thus, Experiment 1 reveals evidence of statistical learning but not of generalization of the learning.

#### Speech production

Figure 2 shows z-score normalized F0 measured from participants’ *beer-pier* speech productions as a function of the Statistical Regularity. Table 2 provides results of the Linear Mixed-Effects Model.

**Table 2:** Experiment 1 speech production F0 across conditions

	*β*	*SE*	*t*	*p*
Intercept	0.01	0.01	1.03	0.302
Test Stimulus F0	0.69	0.04	15.35	< 0.001
Statistical Regularity	0.02	0.03	0.63	0.533
Phoneme Generalization	0.14	0.02	7.05	< 0.001
Dimension Generalization	0.10	0.02	5.10	< 0.001
Test Stimulus F0 x Statistical Regularity	0.16	0.03	5.11	< 0.001
Test Stimulus F0 x Phoneme Generalization	0.26	0.04	6.56	< 0.001
Test Stimulus F0 x Dimension Generalization	0.24	0.04	6.19	< 0.001
Statistical Regularity x Phoneme Generalization	− 0.02	0.04	− 0.47	0.641
Statistical Regularity x Dimension Generalization	− 0.06	0.04	− 1.45	0.148
Test Stimulus F0 x Statistical Regularity x Phoneme Generalization	− 0.44	0.08	− 5.65	< 0.001
Test Stimulus F0 x Statistical Regularity x Dimension Generalization	− 0.53	0.08	− 6.74	< 0.001

Reference levels are Statistical Regularity (Reverse), Test Stimulus F0 (Low F0), Condition (No Generalization). Phoneme Generalization and Dimension Generalization result from simple effects coding comparing the respective conditions to the No Generalization condition

Overall, speech productions elicited by the High (compared to the Low) F0 *beer-pier* test stimuli had higher F0 (t = 15.35, *p* < 0.001). A significant two-way interaction between Test Stimulus F0 and Statistical Regularity (z = 5.11, *p* < 0.001) indicated transfer of statistical learning to production. Simple effects coding comparing production F0 s from the No Generalization Condition to production F0 s from Phoneme Generalization and Dimension Generalization revealed significant main effects of each Condition (Phoneme: z = 7.05, *p* < 0.001; Dimension: z = 5.10, *p* < 0.001). Significant two-way interactions were also evident between each Condition and Test Stimulus F0 (Test Stimulus F0 by Phoneme: t = 6.56, *p* < 0.001; Test Stimulus F0 by Dimension: t = 6.19, *p* < 0.001). As with the perceptual categorization results, two significant three-way interactions indicated that transfer of statistical learning to production differed in both the Phoneme Generalization (t = − 5.65, *p* < 0.001) and Dimension Generalization (t = − 6.74, *p* < 0.001). Conditions relative to the No Generalization Condition. Also similar to perception, the post-hoc analyses only revealed a significant interaction between Test Stimulus F0 and Statistical Regularity in the No Generalization condition (t = 9.44, *p* < 0.001), but not in the Phoneme Generalization (t = 0.87, *p* = 0.383) or Dimension Generalization (t = − 0.82, *p* = 0.410) condition.

The results are clear: perceptual statistical learning across passive exposure failed to generalize in perception. While this replicates the finding of no generalization in the Dimension Generalization (*deer-tear → beer-pier*) condition (Idemaru & Holt, 2014), it contrasts with Phoneme Generalization (*bear-pear → beer-pier*) observed in active tasks that involve trial-by-trial overt speech categorization (Idemaru & Holt, 2020). Naturally, since no generalization was uncovered in perception, transfer of generalization was not seen in production.

## Experiment 2

### Methods

Experiment 1 replicated the null effect of dimension generalization (Idemaru & Holt, 2014) but failed to find evidence of phoneme generalization, contrary to prior reports (Idemaru & Holt, 2020). One interpretation of these results is that statistical learning across passive listening is not sufficient to support generalization. But before this conclusion is drawn, we must rule out the influence of another factor. Recall that in Idemaru and Holt’s (2020) task, participants responded to all tokens, meaning that both the statistics-bearing stimuli and the generalization stimuli were part of the response set. If overlap between exposure and test stimuli is critical for extracting statistics or applying statistics to new stimuli, then a mixed response set should restore phoneme generalization, even with passive exposure. Experiment 2 tested this possibility. First, we aimed to replicate the main findings of statistical learning and its transfer to production, observed in Experiment 1, in a different pair, *bear-pear.* We used this pair as exposure stimuli to test phoneme generalization to a different pair, *beer-pier,* presented in a mixed response set comprised of both *bear-pear* and *beer-pier* tokens with equal frequency.

#### Participants

Based on the power analysis of Experiment 1, we tested 95 participants (48 female) with 87 participants (45 female, M_age_ = 31.3, SD = 6.0 years) entering analyses after application of the Experiment 1 exclusion criteria.

#### Stimuli

Experiment 2 relied on the *beer-pier* and *bear-pear* stimuli from Experiment 1 (Fig. 1A).

#### Procedure

Experiment 2 consisted of six blocks (30 trials each) of trials alternating with Canonical and Reverse regularities. The first two blocks reproduced the No Generalization (*beer-pier→beer-pier*) condition of Experiment 1 (Replication: No Generalization). The remaining four blocks conveyed statistics across *bear-pear* utterances and involved both *bear-pear* (Mixed Response: No Generalization) and *beer-pier* (Mixed Response: Phoneme Generalization) test trials, randomly intermixed such that there was uncertainty about the target of categorization on each trial and the mixed response set involved *beer, pier, bear,* and *pear*. As in Experiment 1, in each block six VOT-unambiguous trials assured online participants’ data quality; no participants were excluded on the basis of responses to these trials. Performance was high and consistent with English regularities (Long VOT, 93%/p/; Short VOT, 88%/b/). Responses from these trials did not enter analyses, resulting in 24 Canonical and 24 Reverse trials for each condition.

#### Statistical analyses

##### Perceptual categorization

The statistical approach was similar to Experiment 1. Our first goal was to replicate statistical learning and its transfer to production in the No Generalization condition, observed in Exp 1. This model included the subset of data from the *beer-pier→beer-pier blocks.* The model included Test Stimulus F0 (High F0, Low F0), Statistical Regularity (Canonical, Reverse) and their interaction, as well as a maximal random effects structure consisting of by-subject random intercept, random slopes for Test Stimulus F0, Statistical Regularity, and the interaction between Test Stimulus F0 and Statistical Regularity over subjects. As in Experiment 1, Statistical Regularity and Test Stimulus F0 fixed effects were center coded (− 0.5 or 0.5).

Next, we examined generalization using blocks with Mixed Response conditions. The model’s dependent variable was coded as voiced (beer, bear) or voiceless (pier, pear). Three fixed effects, Test Stimulus F0 (High F0, Low F0), Statistical Regularity (Canonical, Reverse) and Condition (Mixed Response: No Generalization; Mixed Response: Phoneme Generalization), were included alongside their two-way and three-way interactions. The random-effects structure was similar to the structure used in the Replication task analysis with the addition of a random slope for Condition. All fixed effects were centered coded (− 0.5, or 0.5).

##### Speech production

Acoustic speech analysis followed the Experiment 1 approach with by-participant z-score normalized production F0 s as a continuous dependent variable analyzed with linear mixed-effects models. As with the perceptual categorization analysis, separate models assessed production changes in the Replication and the Mixed Response tasks. Fixed effects and their interactions were identical to those included in the corresponding perceptual categorization models. Both models included by-participant random intercept and random slopes for Test Stimulus F0 and Statistical Regularity. The Mixed Response model also included a random slope for Condition. Neither model tolerated the addition of random slopes for the interaction terms. All fixed effects were center coded (− 0.5 or 0.5).

### Results

#### Perceptual categorization

**Fig. 3:**
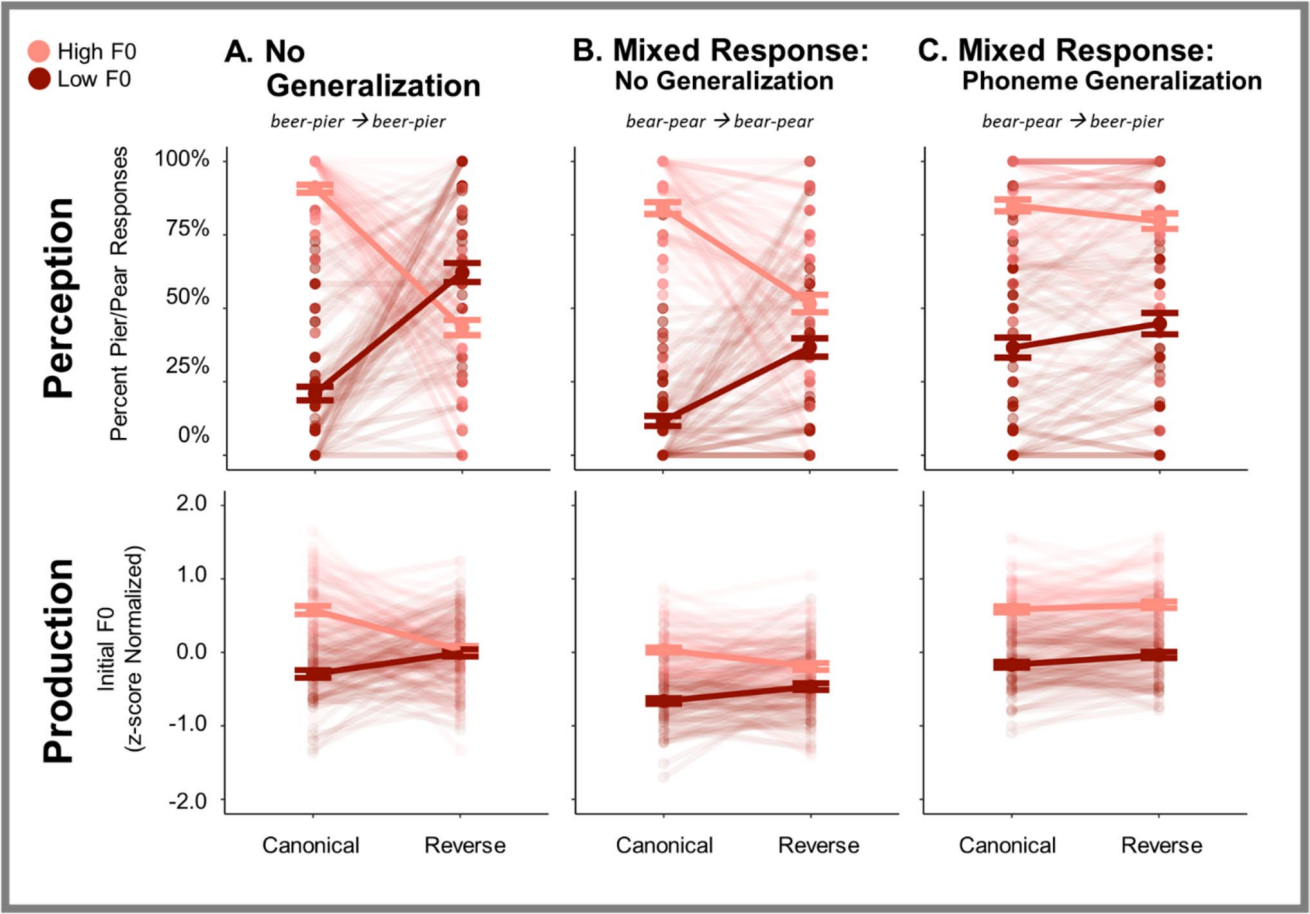
Experiment 2 perception and production results. The top row depicts percent *pier/pear* categorization responses to High and Low F0 *beer-pier* (**A**, **C**) or *bear-pear* (**B**) test stimuli in the context of Canonical and Reverse short-term regularities. The bottom row shows z-score normalized fundamental frequency (F0) speech productions elicited in repetition of these same test stimuli. **A.** Replication: No Generalization (*beer-pier* exposure, *beer-pier* test) is a replication of Experiment 1. **B.** Mixed Response Condition trials with No Generalization (*bear-pear* exposure, *bear-pear* test). **C.** Mixed Response Condition trials requiring Phoneme Generalization (*bear-pear* exposure, *beer-pier* test)

As in Experiment 1, we analyzed perceptual responses for evidence of statistical learning and its generalization to novel tokens (Fig. 3, top row). Analysis of perceptual responses from the Replication task revealed a significant main effect of Test Stimulus F0 (z = 8.18, *p* < 0.001), a significant main effect of Statistical Regularity (z = 2.00, p = 0.045) and, importantly, an interaction between the two (z = 13.87, *p* < 0.001), showing statistical learning in perception.

Table 3 reports the results from the analysis of perceptual response from the Mixed Response blocks. A significant main effect of Test Stimulus F0 indicated that, overall, participants tended to perceive High F0 test stimuli as *pier or pear* and Low F0 as *beer or bear* (z = 18.58, *p* < 0.001). The main effect of Condition was also significant, indicating a difference in/b/versus/p/response rates in the Mixed Response: No Generalization and the Mixed Response: Phoneme Generalization conditions (z = − 5.70, *p* < 0.001). This difference appears to be driven by a bias towards *pier* responses in the Mixed Response: Phoneme Generalization condition, a finding also reported by Idemaru and Holt (2020). A significant two-way interaction between Statistical Regularity and Test Stimulus F0 indicated statistical learning in perception in the Mixed Response blocks (z = 12.50, *p* < 0.001).

**Table 3:** Perceptual categorization of voiced/voiceless test stimuli in mixed response task

	*Β*	*SE*	*z*	*p*
(Intercept)	0.26	0.12	2.18	0.029
Statistical Regularity	0.02	0.08	0.21	0.833
Test Stimulus F0	2.78	0.15	18.58	< 0.001
Condition	− 1.16	0.20	− 5.70	< 0.001
Statistical Regularity x Test Stimulus F0	2.23	0.18	12.50	< 0.001
Statistical Regularity x Condition	0.18	0.13	1.38	0.168
Test Stimulus F0 x Condition	− 0.18	0.14	− 1.23	0.218
Statistical Regularity x Test Stimulus F0 x Condition	2.72	0.27	10.20	< 0.001

Reference levels are Statistical Regularity (Reverse), Target stimulus F0 (Low F0), Condition (Phoneme-Generalization)

There was also a significant three-way interaction between Statistical Regularity, Test Stimulus F0, and Condition (z = 10.20, *p* < 0.001). To unpack this interaction, we fit separate post hoc models to each of the two Conditions, separately. In the Mixed Response blocks, there is evidence of statistical learning in the form of a significant two-way interaction between Statistical Regularity and Test Stimulus F0 in both the No Generalization model (z = 11.47, *p* < 0.001) as well as the Phoneme-Generalization model (z = 3.49, *p* < 0.001).

## Speech production

We next examined transfer of statistical learning to production using z-score normalized F0 measured from *beer-pier* and *bear-pear* productions (Fig. 3, bottom row). First examining the Replication condition, the model reveals the expected main effect of Test Stimulus F0 (t = 9.55, *p* < 0.001), as well as a significant two-way interaction between Test Stimulus F0 and Statistical Regularity indicating the transfer of statistical learning to production (t = 14.55, *p* < 0.001), thereby replicating the transfer observed in Experiment 1.

Table 4 reports the transfer of speech production results from the Mixed Response blocks. Mirroring the perceptual results, the analysis revealed a main effect of Test Stimulus F0 (t = 13.64, *p* < 0.001), as well as a main effect of Condition on production F0 s (t = − 14.81, *p* < 0.001). The latter finding is in line with previous research on intrinsic F0, a tendency for high vowels like the/i/in *beer* to have higher F0 s than low vowels like the/e/in *bear* (Chen et al., 2021; Whalen & Levitt, 1995). Transfer of statistical learning was evident in the significant two-way interaction between Statistical Regularity and Test Stimulus F0 (t = 6.64, *p* < 0.001). There were significant interactions between Statistical Regularity and Condition (t = 3.01, p = 0.003) as well as Test Stimulus F0 and Condition (t = − 6.41, *p* < 0.001).

**Table 4:** Mixed response task productions by test stimulus F0 and condition

	*β*	*SE*	*t*	*p*
(Intercept)	− 0.03	0.01	− 2.34	0.021
Statistical Regularity	− 0.04	0.03	− 1.36	0.177
Test Stimulus F0	0.60	0.04	13.64	< 0.001
Condition	− 0.58	0.04	− 14.81	< 0.001
Statistical Regularity x Test Stimulus F0	0.24	0.04	6.64	< 0.001
Statistical Regularity x Condition	0.11	0.04	3.01	0.003
Test Stimulus F0 x Condition	− 0.24	0.04	− 6.41	< 0.001
Statistical Regularity x Test Stimulus F0 x Condition	0.34	0.07	4.62	< 0.001

Reference levels are Statistical Regularity (Reverse), Target stimulus F0 (Low F0), Condition (Phoneme-Generalization)

Critical for our determining whether generalization transfers to influence speech production, we found a significant three-way interaction between Statistical Regularity, Test Stimulus F0, and Condition (t = 4.63, *p* < 0.001). Post hoc analyses revealed that the two-way interaction between Test Stimulus F0 and Statistical Regularity was significant in the Mixed Response: No Generalization model (t = 8.18, *p* < 0.001) but the perceptual generalization observed for the Mixed Response: Phoneme-Generalization condition did not transfer to production (t = 1.44, p = 0.151).

To summarize, Experiment 2 replicates statistical learning across passive exposure to *beer-pier* and its transfer to speech production. It extends this finding to *bear-pear*, when no generalization is required. Importantly, inclusion of a mixed response set rescued phoneme-level generalization of perceptual statistical learning, although with a smaller magnitude of influence on the generalization pair than the pair experienced across the regularity. This generalization of learning did not transfer to influence speech production.

## General discussion

Does generalization of statistical learning emerge only with learning in an active task? Potentially consistent with this possibility, Wu and Holt (2022) argued that when speech conveys sufficient perceptual information to activate a phonetic category (e.g., via unambiguous VOT), it may generate predictions of the typical mapping of other secondarily diagnostic acoustic dimensions, like F0, to the category representation. In the Reverse condition, these expectations are not met and the mismatch may power error-driven learning that down-weights F0 to minimize future mismatches. Inasmuch as active engagement in a categorization decision might boost category activation, it thus may promote learning and its successful generalization. Yet, Hodson et al. (2023) report statistically equivalent learning outcomes across passive exposure to statistics-bearing speech stimuli and active engagement in a categorization decision across these same stimuli. This latter result suggests that learning across passive exposure may be just as potent as learning across stimuli that demand active categorization. Experiment 2 confirms that statistical learning across passive listening is sufficient to support generalization to stimuli never heard in the accent. Notably, this pattern was not evident in Experiment 1. The difference was that in Experiment 2, a response set included both statistics-bearing and new stimuli with the same initial phoneme. This restored generalization of the learning that accrued across passive listening to the accent.

But why should response set matter? Although speculative, the most reasonable explanation for the influence of response set on generalization may relate to attention and goal-setting, in line with recent findings that show the importance of explicit attentional goals in implicit statistical learning (Zhang & Carlisle, 2023). If participants detect no relationship between exposure and test stimuli, they may tune out exposure stimuli. Under this view, attention is important for learning not because it forces the learner to actively process each statistics-bearing stimulus, but rather because it sets a higher-level behavioral goal in the cognitive-perceptual system. Our results demonstrate the importance of task demands and goals in the context of statistical learning, even when it emerges implicitly across passive exposure. This argues for further research to examine how implicit and explicit task demands influence the nature of information learned across passive exposure.

The present study also lays groundwork for understanding the structure of representation shared between speech perception and production. We replicated the transfer of statistical learning from perception to production reported in Murphy et al. (2024) twice (Experiment 1 and 2, *beer-pier*→*beer-pier*). Additionally, the present work extends evidence of transfer to a novel word pair (Experiment 2, *bear-pear*→*bear-pear*). These results demonstrate that there are rapid and implicit changes to the production system as a result of statistical learning across the patterns of other talkers’ speech. They are interesting, particularly, in light of the finding that most instances of auditory repetition are carried out through the “lexical,” as opposed to the “nonlexical” route (Nozari et al., 2010; Nozari & Dell, 2013). This means that upon hearing a word, the individual retrieves the corresponding stored lexical representation and activates the production chain, rather than simply mapping input to output phonology without fully engaging the production system. In our case, the perceptual judgment performed before production makes it even more likely for participants to use the lexical route. Nevertheless, we observe changes to production. This implies that the results do not reflect a simple imitation of the input. As such, the present data build from Murphy et al. (2024) to provide new insights into phonetic convergence (Pardo et al., 2022) and to extend how other talkers’ speech affects one’s own productions (e.g., Bourguignon et al., 2014, 2016; Lametti et al., 2014).

Yet, even when *bears* affected *beers* in perception, they did not influence production. In Experiment 2, exposure to *bear-pear* distributional regularities led to statistical learning that generalized to *beer-pier* (with a mixed response set). But this learning did not exert an influence on production. The magnitude of generalization (*bear-pear*→*beer-pier*) was smaller than the magnitude of statistical learning across matched trials (*bear-pear*→*bear-pear*), so it is possible that generalization was not robust enough to drive transfer to production. Alternatively, representations subject to learning in perception may differ from those in production, as has been indicated by previous findings that show changes in production can occur independently of changes in perception (Baese-Berk et al., 2024; Kato & Baese-Berk, 2020; Sheldon & Strange, 1982). Future studies of transfer in dimension-based statistical learning are well-poised to address this intriguing possibility because the approach makes it possible to quantify listeners’ and speakers’ detailed reliance on subtle acoustic dimensions, and to manipulate exposure to distributions across them in both passive and active tasks. At this stage, observance of generalization of statistical learning in the absence of transfer to production is important in establishing that production is not simply a mirror of perceptual experience, according with other studies of statistical learning across speech production and perception (e.g., Kittredge & Dell, 2016; Schwartz et al., 2012). Learning-related adjustments to the representations within the production system appear to be necessary.

In conclusion, passive exposure is sufficient to produce generalization of statistical learning in perception, but subtle task demands affect generalization. Inasmuch as the utility of implicit statistical learning over passive exposure is its ability to impact behavior, this highlights how important it will be to direct research toward better understanding how statistical learning statistical learning supports, and is influenced by, task goals and demands.

## Data Availability

The data and tables of the results are available on the OSF (https://osf.io/5uqx8/).

## Code availability

R scripts used for statistical analyses are available on the OSF (https://osf.io/5uqx8/).
